# Supplementary material for: Multimodal imaging maps spinal glymphatic transport in health and its disruption after injury
Source: bioRxiv. 2026 Sep 22:2026.09.16.752122. Preprint. [Version 1] doi: 10.64898/2026.09.16.752122 (PMC13621999; doi:10.64898/2026.09.16.752122)
Supplement: Supplementary file 1 [file NIHPP2026.09.16.752122v1-supplement-1.pdf]

bioRxiv preprint doi: https://doi.org/10.64898/2026.09.16.752122; this version posted September 24, 2026. The copyright holder for this preprint (which was not certified by peer review) is the author/funder, who has granted bioRxiv a license to display the preprint in perpetuity. It is made available under a CC-BY 4.0 International license.

Supplementary Figures

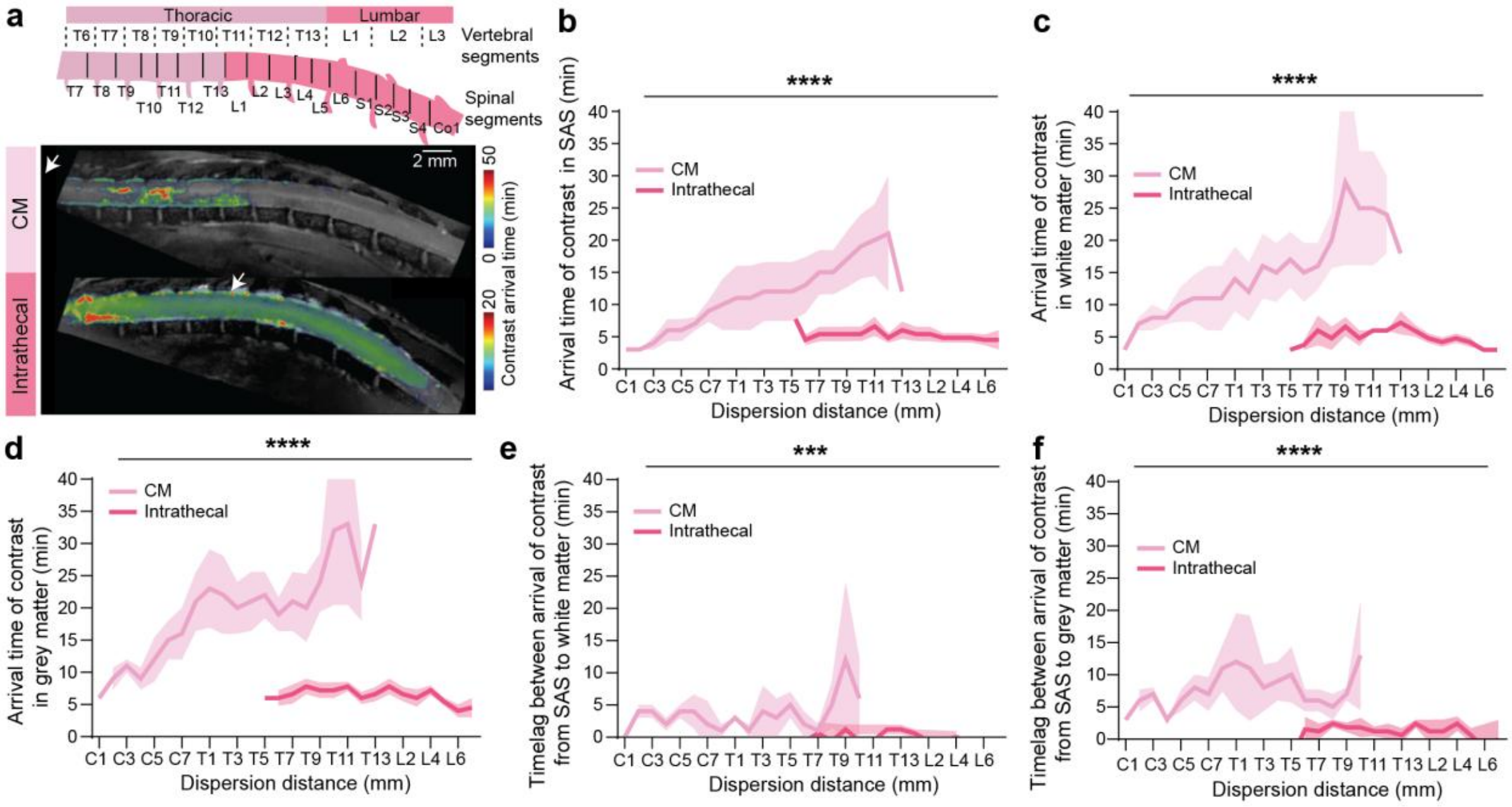

Supplementary Fig. 1 Intrathecal delivery results in faster tracer arrival compared to cisterna magna (CM) injection. a) Example images with segmental sketch illustrating the arrival of contrast agent in the spinal cord SAS, white and grey matter after CM or intrathecal injection. **b)** Contrast agent arrived at the C1 level of the subarachnoid space (SAS) within 3 min after the CM injection and dispersed in the various spinal levels, where it gradually arrived at the T13 level at 12 min (n=3, 2-way ANOVA, P=0.0001). Whereas after the intrathecal injection at L1 level, the contrast arrived bilaterally (at T13 level in 6.0 ± 1.2 min at L2 level in 5.4±1.0 min) and quickly dispersed in the whole thoraco-lumbar spine in an average time of 5.4±0.8 min (n=5). **c)** After CM injection, contrast arrived in white matter (WM) from SAS at C1 level within 3 min where it dispersed along the whole WM and reached at T13 level in 18 min (n=3). After intrathecal injection in L1, it dispersed bilaterally and arrived in WM T13 in 6.0±1.3 min and L2 level in 4.8±0.66 min (n = 5, 2-way ANOVA, P=0.0001). **d)** In grey matter (GM), arrival time was 6 min following CM injection and 6.6±1.1 min at T13 and 6.6±1.0 min at L2 level following intrathecal delivery at L1 spinal level (n = 5, 2-way ANOVA, P=0.0001). **e, f)** After CM injection, average time lag between the arrival of contrast in WM and GM from SAS was 3.4±0.54 min and 8.3±0.9 min, respectively. However, after intrathecal injection the average time lag for WM and GM from SAS was 0.6±0.19 min and 1.7±0.17 min, respectively (CM vs intrathecal control, 2-way ANOVA, WM: P=0.001; GM: P=0.0001).

bioRxiv preprint doi: https://doi.org/10.64898/2026.09.16.752122; this version posted September 24, 2026. The copyright holder for this preprint (which was not certified by peer review) is the author/funder, who has granted bioRxiv a license to display the preprint in perpetuity. It is made available under a CC-BY 4.0 International license.

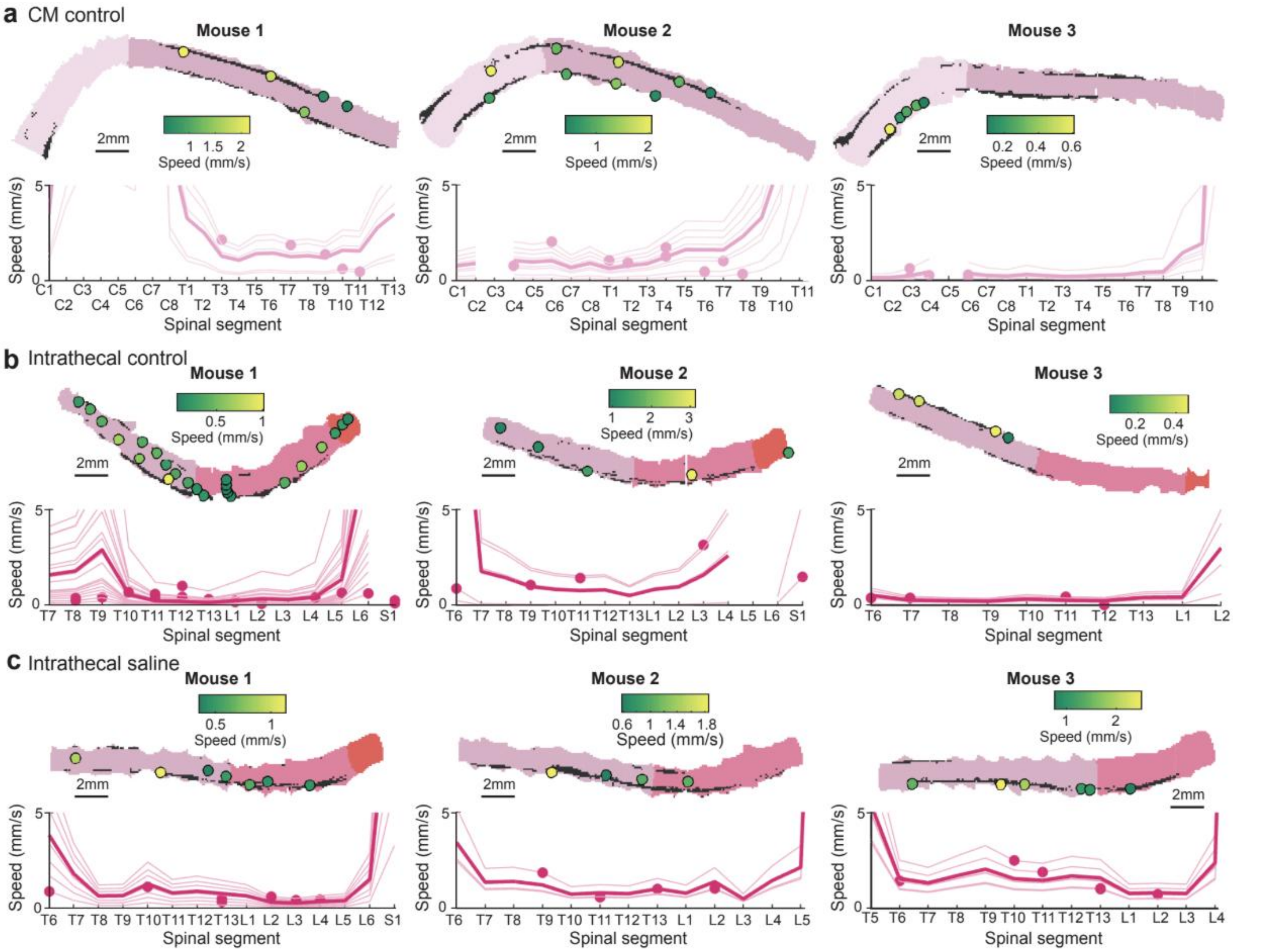

Supplementary Fig. 2 SAS volumetric analysis and speed measurements. a) a-c) Individual mouse data of contrast agent segment-wise speed calculation in the SAS after CM (a) or intrathecal (b-c) injection.

bioRxiv preprint doi: https://doi.org/10.64898/2026.09.16.752122; this version posted September 24, 2026. The copyright holder for this preprint (which was not certified by peer review) is the author/funder, who has granted bioRxiv a license to display the preprint in perpetuity. It is made available under a CC-BY 4.0 International license.

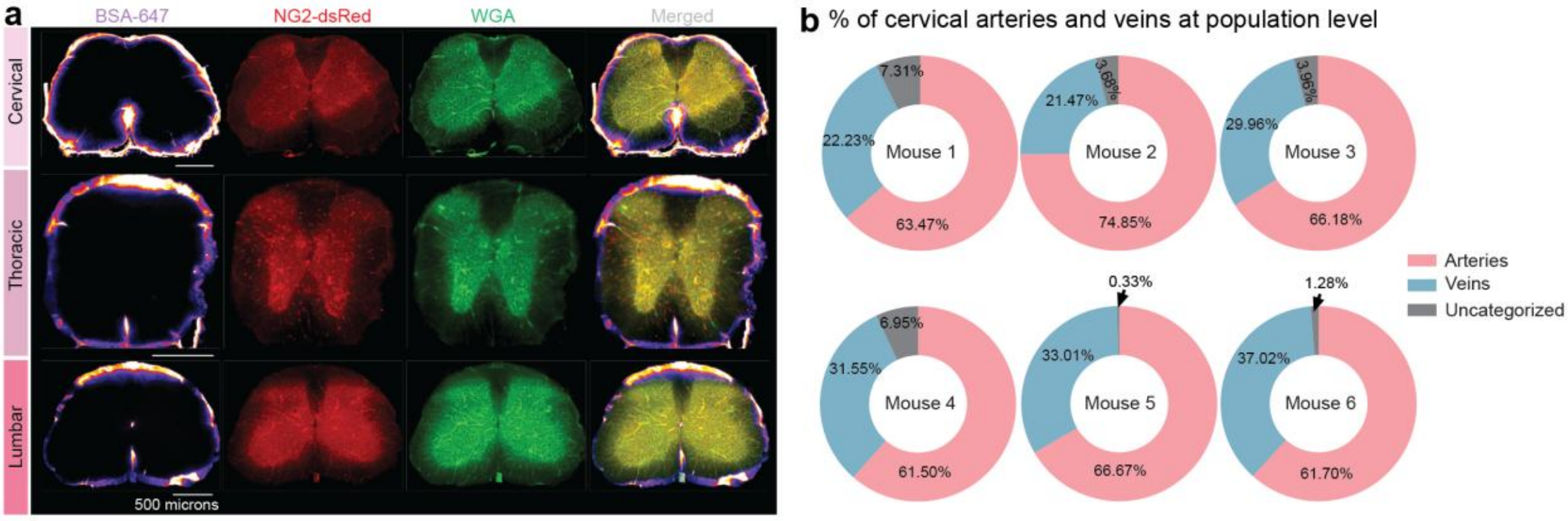

Supplementary Fig. 3 Cisterna Magna infused tracer predominantly flows along arterial perivascular spaces. a**)** Example images show spinal cord vessels labelled by bovine serum albumin conjugated with Alexa Fluor 647 (BSA^{647}) and labelling of arteries and veins by wheat germ agglutinin conjugated with Alexa Fluor 488 (WGA^{488}) in NG2-dsRed mice at cervical, thoracic and lumbar spinal cord levels. **b)** % of vessels in the cervical spinal cord at the population level (14-20 spinal sections per mouse) in each mouse spinal cord showed maximum % of arteries (in red) were present in the cervical region, followed by veins (in cyan) with the minimum percentage of uncategorized vessels (in grey).

bioRxiv preprint doi: https://doi.org/10.64898/2026.09.16.752122; this version posted September 24, 2026. The copyright holder for this preprint (which was not certified by peer review) is the author/funder, who has granted bioRxiv a license to display the preprint in perpetuity. It is made available under a CC-BY 4.0 International license.

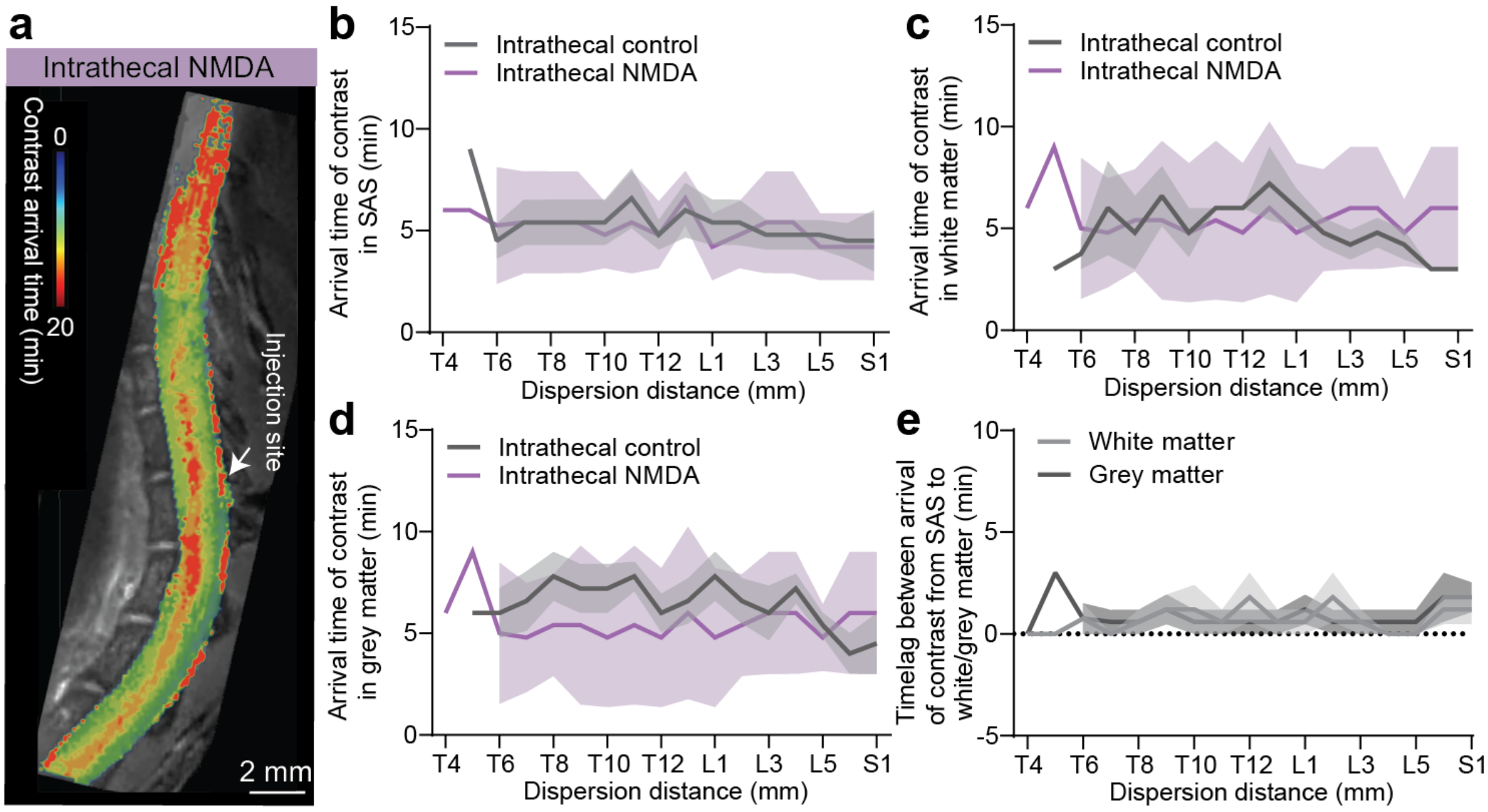

Supplementary Fig. 4 NMDA injection did not change the arrival of contrast in the spinal cord. a) Example image showing arrival and dispersion of contrast delivered intrathecally 24 h after NMDA injection. **b)** After intrathecal injection at L1, bilateral arrival and dispersion of contrast occurred where the contrast arrived and dispersed in the SAS quickly (rostrally at T13 within 6.0 min and caudally at L2 spinal level within 4.8±0.66 min) after SCI, which was similar to the intrathecal control conditions (n=5). **c, d)** After SCI, the contrast arrived at WM within 6.0±1.47 min at T13 level and caudally within 6.0±1.2 min at L2 level and in GM T13 level within 6.0±1.69 min and caudally at L2 level within 5.4±1.0 min (n=5). **e)** Time lag of contrast SAS to entry into the WM and GM after SCI was 0.9±0.17 min and 0.93±0.16 min, respectively (n=5). Note that no significant difference was observed in the intrathecal control (n=5) and SCI conditions in all cases.

bioRxiv preprint doi: https://doi.org/10.64898/2026.09.16.752122; this version posted September 24, 2026. The copyright holder for this preprint (which was not certified by peer review) is the author/funder, who has granted bioRxiv a license to display the preprint in perpetuity. It is made available under a CC-BY 4.0 International license.

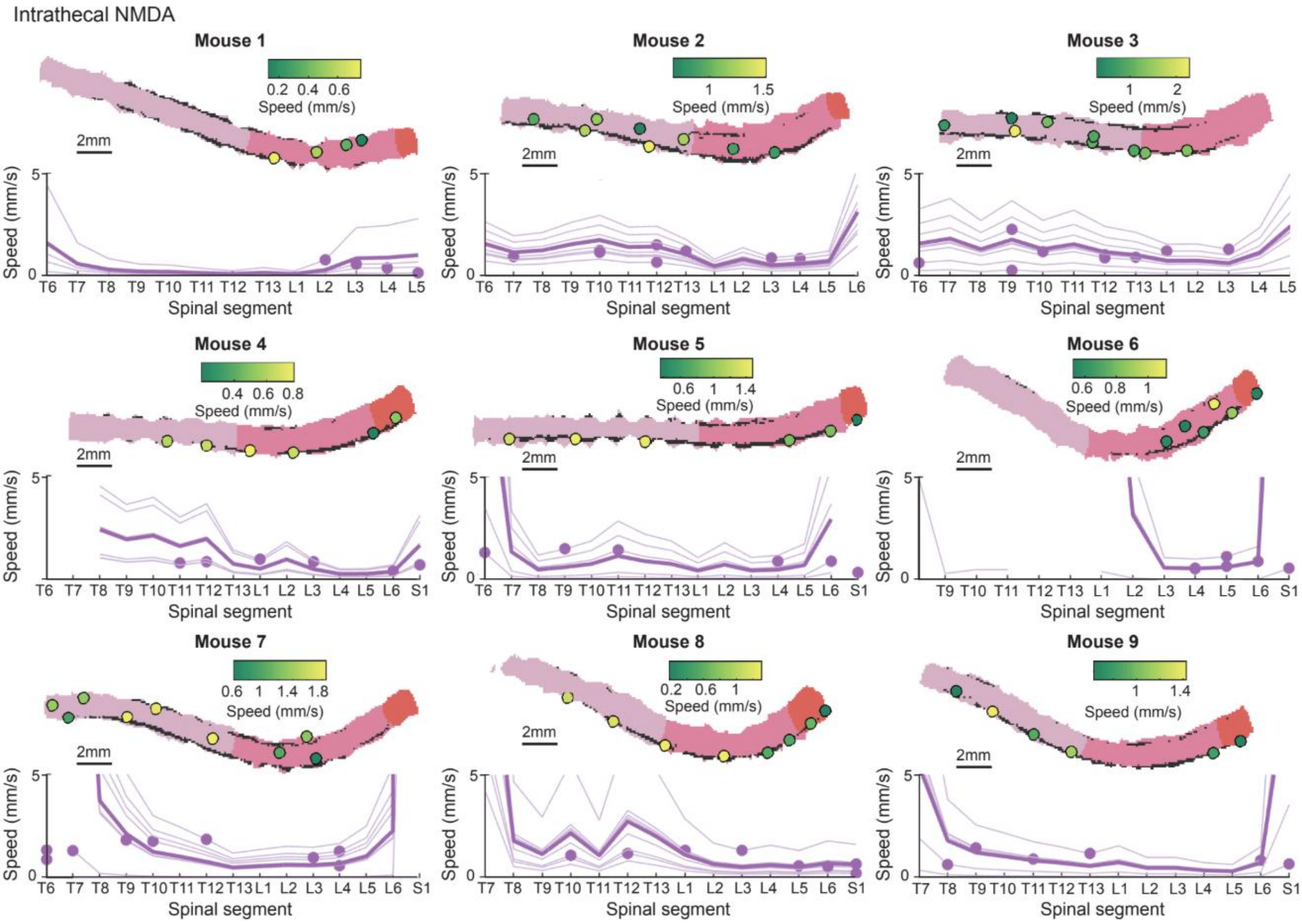

Supplementary Fig. 5 Speed measurement of contrast agent (delivered intrathecally) in the SAS 24 h after the NMDA injection. Individual mouse data from the SAS showing changes in contrast agent speed in each spinal segment after the lesion.
